# Computerized Capability of Office-Based Physicians to Identify Patients Who Need Preventive or Follow-up Care — United States, 2017

**DOI:** 10.15585/mmwr.mm6944a2

**Published:** 2020-11-06

**Authors:** Damon F. Ogburn, Brian W. Ward, Alicia Ward

**Affiliations:** ^1^Division of Health and Nutrition Examination Survey, National Center for Health Statistics, CDC; ^2^Division of Health Care Statistics, National Center for Health Statistics, CDC; ^3^Strategic Innovative Solutions, St. Petersburg, Florida.

Preventive care or follow-up care have the potential to improve health outcomes, reduce disease in the population, and decrease health care costs in the long-term (*1*). Approximately one half of persons in the United States receive general recommended preventive services (*2*,*3*). Missed physician appointments can hinder the receipt of needed health care (*4*). With electronic health record (EHR) systems able to improve interaction and communication between patients and providers (*5*), electronic reminders are used to decrease missed care. These reminders can improve various types of preventive and follow-up care, such as immunizations (*6*) and cancer screening (*7*); however, computerized capability must exist to make use of these reminders. To examine this capability among U.S. office-based physicians, data from the National Electronic Health Records Survey (NEHRS) for 2017, the most recent data available, were analyzed. An estimated 64.7% of office-based physicians had computerized capability to identify patients who were due for preventive or follow-up care, with 72.9% of primary care physicians and 71.4% of physicians with an EHR system having this capability compared with surgeons (54.8%), nonprimary care physicians (58.5%), and physicians without an EHR system (23.4%). Having an EHR system is associated with the ability to send electronic reminders to increase receipt of preventive or follow-up care, which has been shown to improve patient health outcomes (*8*).

NEHRS is a nationally representative, mixed-mode survey of U.S. office-based physicians that collects information on the adoption, use, and interoperability of EHR systems. Information on physician and practice characteristics is also collected. NEHRS is sponsored by the Office of the National Coordinator for Health Information Technology and conducted by CDC’s National Center for Health Statistics (NCHS) annually as a sample survey of nonfederally employed, office-based physicians who are primarily engaged in direct patient care and are located in the 50 U.S. states or the District of Columbia. Physicians with a primary specialty of anesthesiology, pathology, or radiology are excluded. The 2017 NEHRS had a sample of 10,302 physicians and a weighted response rate of 33.6%.* Physicians were identified as having computerized capability to identify patients who are due for preventive or follow-up care if they responded affirmatively to the question, “Does the reporting location use a computerized system to identify patients due for preventive or follow-up care?” Having this computerized capability indicates that the physician’s office uses a software program that identifies if a patient is in need of preventive or follow-up care, and if so, has a computer send an alert or reminder to notify the patient that this care is needed (*8*). This capability is distinct from an EHR system, which might contain medical information from all clinicians involved in a patient’s care (not just those in a specific office), and all authorized clinicians involved in a patient’s care can access the information contained. However, these computerized notifications might be part of some EHR systems.

The percentage of physicians having computerized capability to identify patients due for preventive or follow-up care was estimated for U.S. office-based physicians by selected physician characteristics, including specialty, medical degree, sex, age group in years, currently accepting new patients, and practice characteristics (size, ownership, uses an EHR system, and metropolitan status). The prevalence ratios of physicians having this computerized capability (adjusted for the above-mentioned physician and practice characteristics) were also examined by these characteristics using multivariable logistic regression. The estimates resulting from this regression were used to calculate prevalence ratios according to methods detailed elsewhere (*9*). All estimates meet NCHS standards of reliability for proportions (*10*). Sample weights were used for all analyses, and NEHRS complex sample design was accounted for by using SUDAAN software (version 11.0.1; RTI International). For comparisons of estimates among subgroups, statistical significance (p<0.05) was determined by two-tailed significance tests. All reported differences between subgroups were statistically significant.

In 2017, 64.7% of U.S. office-based physicians had the computerized capability to identify patients due for preventive or follow-up care (Table). A higher percentage of primary care physicians (72.9%) had this computerized capability than did surgeons (54.8%) and other nonprimary care physicians (58.5%). Seventy percent of physicians aged 45–54 years had this capability compared with 57.2% of those aged 65–84 years. No differences were found by medical degree, sex, currently accepting new patients, and metropolitan status.

**TABLE:** Percentages and adjusted prevalence ratios for office-based physicians who have computerized capability to identify patients who are due for preventive or follow-up care, by selected physician and practice characteristics — National Electronic Health Records Survey, 2017

Characteristic	% (95% CI)	aPR (95% CI)
**Total**	**64.7 (61.5–67.8)**	**—**
**Physician characteristic**		
**Specialty**		
Primary care	72.9* (68.6–77.0)	Ref*
Surgical care	54.8^†^ (47.3–62.0)	0.8^†^ (0.7–0.9)
Nonprimary care	58.5^†^ (52.5–64.4)	0.8^†^ (0.8–0.9)
**Medical degree**		
Doctor of medicine	64.8 (61.4–68.0)	Ref
Doctor of osteopathic medicine	63.6 (50.7–75.3)	1.0 (0.8–1.2)
**Sex**		
Female	67.1 (61.4–72.5)	Ref
Male	63.7 (59.8–67.5)	1.0 (0.9–1.1)
**Age group (yrs)**		
<45	67.2 (60.0–73.9)	Ref
45–54	70.0^§^ (64.3–75.4)	1.1 (1.0–1.2)
55–64	63.8 (58.1–69.2)	1.1 (0.9–1.2)
65–84	57.2^§^ (49.1–65.0)	1.1 (0.9–1.3)
**Currently accepting new patients**		
Yes	65.3 (62.1–68.5)	1.1 (0.9–1.3)
No	58.8 (44.3–72.1)	Ref
**Practice characteristics**		
**Size**		
Solo practice	53.1^¶^ (46.3–59.9)	Ref
2 physicians	70.2** (61.9–77.6)	1.1 (0.9–1.3)
3–5 physicians	66.8** (60.5–72.7)	1.0 (0.9–1.1)
≥6 physicians	69.6** (64.5–74.4)	1.0 (0.9–1.1)
**Physician/Physician group ownership**		
Yes	61.0^††^ (56.8–65.1)	0.9 (0.8–1.0)
No	70.2^††^ (65.1–75.0)	Ref
**Uses EHR system**		
Yes	71.4^††^ (68.3–74.4)	2.9^††^ (2.0–4.4)
No	23.4^††^ (15.3–33.3)	Ref^††^
**Metropolitan status**		
Metropolitan statistical area (MSA)	64.7 (61.3–67.9)	Ref
Non–MSA	64.8 (54.3–74.3)	1.0 (0.9–1.1)

**Abbreviations**: aPR = adjusted prevalence ratio; CI = confidence interval; EHR = electronic health record; Ref = referent.

* Significant difference compared with surgical care and nonprimary care.

^†^ Significant difference compared with primary care.

^§^ Significant difference between physicians aged 45–54 and 65–84 years.

^¶^ Significant difference compared with two physicians, three to five physicians, and six or more physicians.

** Significant difference compared with solo practice.

^††^ Significant difference between yes and no.

A lesser percentage of physicians in solo practice (53.1%) had this computerized capability than did physicians at practices with two physicians (70.2%), three to five physicians (66.8%), and six or more physicians (69.6%). A higher percentage of physicians at practices that were not owned by a physician/physician group (70.2%) had this computerized capability compared with those at practices that were owned by physicians (61.0%). A higher percentage of practices that used an EHR system (71.4%) than did not use an EHR system (23.4%) had this computerized capability.

When accounting for physician and practice characteristics simultaneously in a logistic regression model, only few statistically significant differences remained. The proportion of surgeons (adjusted prevalence ratio [aPR] = 0.8; 95% confidence interval [CI] = 0.7-0.9) and other nonprimary care physicians (aPR = 0.8; 95% CI = 0.8-0.9) having the computerized capability to identify patients due for preventive or follow-up care was lower than the proportion of primary care physicians. In addition, the proportion of physicians at practices that used an EHR system was approximately three times greater for having this computerized capability compared with physicians at practices that did not use an EHR system (aPR = 2.9; 95% CI = 2.0–4.4).

## Discussion

Results show that the percentage of U.S. office-based physicians with computerized capability to identify patients for preventive or follow-up care is higher among certain physician and practice characteristics, including a physician’s specialty, age, practice size, ownership, and use of an EHR system. However, when controlling for these characteristics simultaneously through multivariable analyses, only physician specialty and use of an EHR system had a significant association with this capability: a lower proportion of surgeons and other nonprimary care physicians had this capability than primary care physicians, while a higher proportion of physicians whose practice used an EHR system had this capability compared with physicians at a practice without an EHR system. Because this computerized capability can be included in some EHRs (*8*), this finding might be expected.

The findings in this report are subject to at least three limitations. First, because of the scope, the data analyzed only included nonfederal, office-based physicians, and therefore the ability to examine the computerized capability to identify patients for preventive or follow-up care by physicians in hospitals, jails and prisons, Veterans Affairs medical facilities, or other non-office-based locations could not be determined. Second, only having this computerized capability could be examined, not whether the physician regularly used it, or whether it was effective in getting the patient to make a care appointment. Finally, there might also be additional physician characteristics (e.g., years in practice) and practice characteristics (e.g., daily patient volume) that could be considered but were not available in NEHRS.

Previous research indicates that the use of electronic reminders can increase the likelihood of patients returning for preventive or follow-up care (*6*,*7*). However, before this can occur, a physician must have the capability to identify these patients. Having an EHR system can increase the likelihood a physician has this capability, potentially increasing the potential for patient returns for preventive or follow-up care through use of electronic reminders. This has been shown to improve patient health outcomes (*8*).

**Summary**

**What is already known about this topic?**

Preventive or follow-up care can improve health outcomes and reduce disease. Missed physician appointments hinder receipt of health care. Electronic reminders can reduce missed appointments.

**What is added by this report?**

In 2017, 64.7% of U.S. office-based physicians had computerized capability to identify patients due for preventive or follow-up care. A lower percentage of surgeons and nonprimary care physicians had this capability compared with primary care physicians. A higher percentage of physicians whose practice used an electronic health record system had this capability.

**What are the implications for public health practice?**

These findings highlight physician and practice characteristics associated with capability for computerized identification of patients due for preventive or follow-up care which might inform efforts to increase patient follow-up.

## Footnotes

* A copy of the NEHRS questionnaire is available at https://www.cdc.gov/nchs/data/ahcd/2017_NEHRS_Sample_Card.pdf. Data from the 2017 NEHRS are available at https://www.cdc.gov/rdc/index.htm.
